# Effect of Chronic Moderate Caloric Restriction on the Reproductive Function in Aged Male Wistar Rats

**DOI:** 10.3390/nu14061256

**Published:** 2022-03-16

**Authors:** Pablo López de Jesús, Edith Arenas-Ríos, Mirna Ruíz-Ramos, Juan Carlos Flores-Alonso, Víctor Manuel Mendoza-Núñez, Isabel Arrieta-Cruz, Marcela Arteaga-Silva

**Affiliations:** 1 Doctorado en Ciencias Biológicas y de la Salud, Universidad Autónoma Metropolitana, Ciudad de México 09340, Mexico; pldj_89@hotmail.com; 2 Departamento de Biología de la Reproducción, Universidad Autónoma Metropolitana-Iztapalapa, Ciudad de México 09340, Mexico; editharenas2000@yahoo.com.mx; 3 Unidad de Investigación en Gerontología, Facultad de Estudios Superiores Zaragoza, Universidad Nacional Autónoma de México, Ciudad de México 09230, Mexico; mirna1411@yahoo.com.mx (M.R.-R.); mendovic@unam.mx (V.M.M.-N.); 4 Centro de Investigación Biomédica de Oriente, Instituto Mexicano del Seguro Social, Hospital General de Zona No. 5, Puebla 74360, Mexico; flores_alonso_jc@hotmail.com; 5 Departamento de Investigación Básica, Instituto Nacional de Geriatría, Secretaria de Salud, Ciudad de México 10200, Mexico; iarrieta@inger.gob.mx

**Keywords:** calorie restriction, male sexual behavior, seminiferous tubules, testis, testosterone

## Abstract

Caloric restriction (CR) has been shown to be an effective nutritional intervention for increasing longevity in some animal species. The objective of this study was to evaluate CR’s effects on metabolic and reproductive parameters in 12-month-old male Wistar rats. The rats were distributed in three groups: control, CR at 15%, and CR at 35% for 6 (up to 18 months of age) and 12 months (up to 24 months of age). At the end of CR treatment, we evaluated reproductive (male sexual behavior (MSB), sperm quality) and biochemical parameters (plasma glucose, glucose-regulating hormone, and sex steroid levels), and quantified annexin V in the seminiferous epithelium. Results showed that MSB and sperm quality were improved after 6 months of CR associated with increases in plasma testosterone and decrease annexin V in the seminiferous epithelium of the testicles compared to their control group. The metabolic profile of the CR rats also improved compared to controls. However, these effects of CR on reproductive parameters were not maintained after 12 months of CR. Findings suggest that beginning CR at the age of maturity reestablishes the behavioral sexual response and reproductive function in older animals after 6 months of CR and improves endocrine functioning during aging.

## 1. Introduction

Several diseases, such as cancer and cardiovascular, metabolic, and neurodegenerative illnesses, have a common risk factor: aging. This process is characterized for molecular and cellular dysfunction on several levels, for example, telomere attrition, cellular senescence, loss of protein homeostasis, accumulation of damaged mitochondria, epigenetic modifications, increase of pro-inflammatory markers, and decrease in hormone activity [1,2]. All these cellular changes during aging lead to progressive decline of physiological functions in species, including the reproductive system. On the other hand, efforts to reduce the negative impact of aging have incorporated nutritional modifications, such as healthy diet, diet enriched in polyphenols and polyunsaturated fatty acids, or caloric restriction (CR) [3].

CR refers to reducing the ingestion of calories without detriment to nutrition. It involves decreasing all the nutrients that an individual consumes (proteins, lipids, minerals, carbohydrates), so it affects only energy ingestion and does not compromise the organism’s functioning [4,5]. CR is one of the most widely studied and effective gero-protective interventions in diverse animal models, including *Caenorhabditis* *elegans*, *Drosophila melanogaster*, and mice [6,7]. CR activates several molecular signaling pathways, such as SIRT1 (member of the sirtuin family), mTOR (mammalian Target of Rapamycin), IGF1 (Insulin-like growth factor I), PGC-1α (peroxisome proliferator-activated receptor gamma coactivator 1-alpha), FOXO3 (forkhead Box O3), and AKT (*protein* kinase B), which are capable of modulating aging and prolonging life in these species [8,9].

Growing evidence suggests that CR has advantages for the lengthening of life and health of primates and humans [10,11,12]. Indeed, studies have demonstrated that CR can enhance physical condition, reduce body mass index, and improve skin condition and hepatic and renal functions while reducing the risk of cardiovascular disease. It may also participate in maintaining endocrine function and reestablishing sexual behavior [3,5,6,10]. In rodents, some observed effects of CR have been in the immune system by improving the lymphocyte viability and proliferation and increasing the release of anti-inflammatory (IL-10) cytokines and in the cardiometabolic system, where the glucose and lipids profile were ameliorated, and oxidative stress was reduced [13,14]. Interestingly, a recent study that used transcriptomic analysis on rat liver with short-term or mild-to-moderate CR showed mainly seven genes (*Nampt*, *Glul*, *Sult1a1*, *Fmo1*, *Cmtm6*, *Hsp90aa1*, and *Actg1*) that could be relevant for the beneficial effects of CR and might be used as sensitive biomarkers to evaluate the impact of nutritional intervention [15].

Ample evidence indicates that CR is an efficacious nutritional intervention to reduce weight through the modification of adipokines, myokines, and cardiokines in the skeletal muscle and adipose tissues. For example, CR increases leptin and adiponectin secretion and mitochondrial function and improves sensitivity to insulin. Recently studied, the apelin hormone is an adipomyokine that has a protective role in fat, heart, and skeletal muscle tissues, and some studies have tested its relationship with the beneficial effects of CR, observing that apelin secretion is diminished in obese subjects and helps to reduce insulin resistance. In the case of sex steroid hormones, CR has modified the testosterone (T)/estradiol (E_2_) ratio as well as anti-inflammatory properties in their target tissues. Furthermore, CR helps to improve reproductive health in mammals [16,17]. Adequate levels of hormones, such as T, E2, and prolactin (PRL), are necessary for the expression of male sexual behavior (MSB) in mammals, which occurs in response to the presence of a potential sex partner [18]. Performance of MSB requires a series of external and internal factors, such as the circadian or seasonal cycle, temperature, and adequate functioning of the hypothalamic-pituitary-gonadal (HPG) axis [18,19], as well as a neural substrate that is capable of receiving and processing external stimuli to initiate the sexual interaction that generates motor responses: mounting, intromission, and ejaculation [20,21,22]. Any alteration of these hormone levels can inhibit MSB [23,24,25]. Ample evidence demonstrates that MSB decreases with age in male rats [26,27] by altering the parameters of latency to ejaculation and intromission indices in rats aged 24 months [28], and by producing a deficit in testicular functional [29]. In consequence, reports have verified reductions in the number of spermatozoids and germinal cells. This loss could be related to a decrease in the capacity of the Sertoli cells to sustain the survival and differentiation of those cells or possibly a reduction in the availability of T produced by Leydig cells [30]. Studies in aged rats showed a reduction in the sperm quality and MSB [28]; in the human case, aging produces alterations in sexual desire, erectile function, and sexual activity [29]. This evidence suggests that aging may be a determining factor in the progressive reduction of the male reproductive function.

At present, the beneficial effects of CR on the sexual behavior of rodents have not been clarified because effects both positive and negative have been observed depending on the percentage of reduction of nutrients, the time of exposure to CR, and the age at the onset of CR [31]. For example, male rats in reproductive age subjected to CR at 50% for 4–5 weeks had significant reductions in body weight, deficient MSB performance, and reduced sexual preference for females, all associated with lower T concentrations. In contrast, rats subjected to CR at 25% did not present any of those alterations [32]. Results of this nature indicate the need to inquire more deeply into the effects of chronic, moderate CR on the execution of MSB in diverse stages of the aging process, so the objective of this study was to determine the long-term effect of chronic moderate CR on the reproductive parameters of older Wistar rats.

## 2. Materials and Methods

### 2.1. Animals

A total of 27 male rats of the Wistar strain aged 12 months old were utilized. They were housed individually at the animal care center of the Universidad Autónoma Metropolitana-Iztapalapa and maintained under a 12 h light-dark cycle with lights off at 08:00 a.m., a temperature of 24 ± 1 °C, and ad libitum access to water and food. The animals had access to regular chow during fifteen days before starting CR (Bio-Dieta-Lab DDL-7100 ABENE, Mexico). The net energy obtained for regular chow intake was 117–164 Kcal/d (29.47 ± 3.86 food grams). In the CR groups, the net energy was 95–104 Kcal/d (23.33 ± 0.81 food grams) for 15% of CR and 77–87 Kcal/d (19.33 ± 0.81) for 35% of CR (Bromatological analysis was performed by Facultad de Medicina Veterinaria y Zootecnia de Universidad Nacional Autónoma de México). All handling and experimental procedures complied with international regulations (NIH, 2011) and Mexico’s Official Standard (NOM-062-ZOO-1999, reviewed in 2001) for the use and care of laboratory animals.

### 2.2. Experimental Design

**Figure 1:**
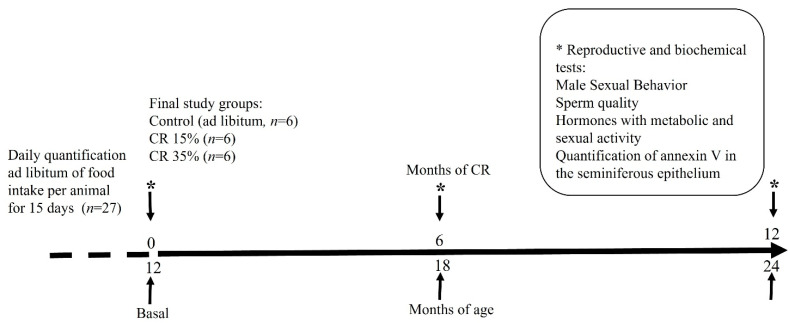
Timeline of the experimental design. Food intake ad libitum per animal was recorded for 15 days before CR (discontinuous line). The experiment began when the rats reached 12 months old (basal 0) with three groups: control (food ad libitum), CR at 15%, and CR at 35% (*n* = 6 for each group, final study). Reproductive and biochemical tests were performed (*) at baseline and 6 and 12 months of CR.

Figure 1 shows the temporal sequence of the experiments conducted. Fifteen days before beginning, the animals were housed individually to allow daily ad libitum quantification of food intake. The animals were then subjected to two CR groups: 15% and 35% CR according to their daily food intake. The animals with CR were fed once a day at 09:00 a.m., and food was available until depletion. The experimental procedure began when the animals reached 12 months old (basal, time 0) with three groups: control (food ad libitum), CR at 15%, and CR at 35% during 6 or 12 months of CR. The study of survival was carried out with all 27 animals; however, due to the death of some animals during the study from age complications, the final number of animals per group was maintained to six animals at the end of the study by adding new rats with similar experimental conditions. The rats that survived to the end of the protocol were subjected to the following reproductive and biochemical tests: MSB, sperm quality, and levels of sex hormones and hormones with metabolic activity. The animals were fasted and sacrificed at the end of the experiment, and blood and tissue samples were obtained and stored for further analysis.

### 2.3. Body Weight during Caloric Restriction and Survival

The body weight of all rats in the three study groups was recorded daily throughout the nutritional intervention. To determine survival rates, we recorded the number of rats that died each three months [33].

### 2.4. Assessment of Sexual Behavior and Sperm Quality

For testing, a male was placed in a plexiglass arena (45 cm in diameter) 5 min before a receptive female rat was introduced as a stimulus. Females of the same strain were rendered sexually receptive by subcutaneous injections of 5 μg/50 μL of estradiol benzoate and 1 mg/100 μL of progesterone (both from Sigma Chemical, Co., St. Louis, MO, USA), administered 48 and 4 h before testing, respectively. Test duration was 30 min after presenting the female. The sexual behavior tests were performed at 7-day intervals under red lights 3 h after onset of the dark phase. The following parameters were recorded: latencies to first mount, first intromission, first ejaculation, and the number of mounts (with pelvic thrusting) and intromissions (mounts with pelvic thrusting and penile insertion) during the first copulatory series. In addition, ejaculation frequency (number of ejaculations during 30 min of recording) and post-ejaculatory intervals (time between ejaculation and subsequent intromission) were registered. The hit rate was the ratio between the number of intromissions and the number of mounts + intromissions (for a detailed description of the MSB parameters in 14). To evaluate the sperm parameters, spermatozoids were obtained from the cauda of the epididymis by perfusion with 1 mL of PBS, and then, 10-μL aliquots were taken to determine motility and vitality values. For the parameter of sperm concentration, dilutions corresponding to WHO guidelines were performed and adequately modified for rats [34]. DNA fragmentation analysis was performed following the previously reported methodology [35], adjusting the incubation time in the alkaline lysis buffer to 90 min.

### 2.5. Determination of Plasma Glucose and Hormone Levels

All the rats were anesthetized with ketamine (50 mg/kg) and xylazine (10 mg/kg), administered intraperitoneally in order to obtain blood samples by cardiac puncture. Each sample was placed in a tube with serum-separator gel (REF 36815, BD Vacutainer SST, México). Serum was recovered by centrifugation (3000 rpm, 15 min). Glucose levels were measured by the glucose oxidase method in a Merck Vitalab Eclipse autoanalyzer (Merck, Dieren, The Netherlands). The commercial ELISA kit (DRG International Inc., NJ, USA) was utilized to determine the sex hormones: testosterone (EIA-1559), estradiol (EIA-2693), and prolactin (EIA-1291). For the other hormones, namely insulin (80-INSRT-E01), glucagon (48-GLUHU-E01), leptin (22-LEPMS-E01), and adiponectin (22-ADPRT-E01), a different commercial ELISA kit was used (Alpco, Bensheim, Germany). Each serum sample was assayed in duplicate and read at 450 nm in a Multiskan™ GO Thermo Scientific multiplate spectrophotometer (Waltham, MA, USA).

### 2.6. Testicular Apoptosis Analysis

The rats were infused by intra-cardiac procedure with 4% paraformaldehyde, and the testes processed for embedding in paraffin. Tissue sections were obtained with a Leica RM2155 microtome at a thickness of 5 µm. Detection of annexin V was performed using an Alexa fluor^TM^ 488 Annexin V/Dead cell Apoptosis kit (Thermo Fisher Scientific, Eugene, Oregon, USA). Histological analyses were carried out using an epifluorescence microscope equipped with an Axio Observer 2 Apotome V2-coupled structured lighting system (Carl Zeiss, Germany). A total of 20 transverse sections of the seminiferous tubules were analyzed per animal by the annexin V technique described previously [36]. The area of the seminiferous epithelium was determined by subtracting the internal area from the external area using a ZEN black image-analyzing system (Karl Zeiss, Germany).

### 2.7. Statistical Analyses

Results are presented as mean ± SE. The survival analysis was performed with the Kaplan–Meier test. Data on body weight, MSB, sperm quality parameters, T, E_2_, prolactin, and serum metabolic markers were analyzed using two-way analysis of variance tests (ANOVA) followed by Tukey’s with the GraphPad Prism 8.0 statistical program (La Jolla, CA, USA). The criterion for statistical significance was *p* < 0.05.

## 3. Results

### 3.1. Body Weight during Caloric Restriction and Survival

After 6 and 12 months of CR 15% and CR 35%, the rats quickly lost weight, and a significantly reduction was shown compared to control groups (*p* < 0.01), and there was also a significant increase in the control group compared to the basal value (*p* < 0.05, Table 1).

**Figure 2:**
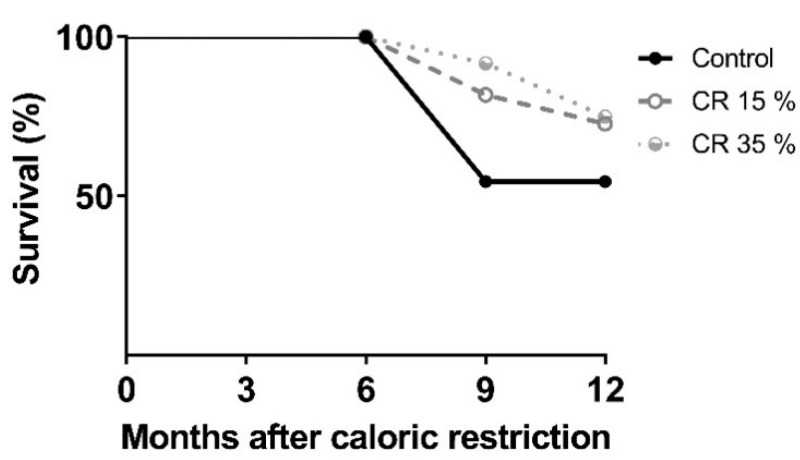
Effect of CR on survival in aged male Wistar rats. The values obtained show percentage of survival of the rats with CR at 15% or 35% for 6 and 12 months of CR.

The evaluation of survival showed 100% of the rats in the CR 15% and 35% groups remained alive after 6 months. At 9 months, 50% of the rats in the control group had survived compared to 100% and 80%, respectively, for the CR 15% and 35% groups. After 12 months of CR, survival in the control group remained at 50% and 75% of survival in both CR 15% and 35% groups (Figure 2).

### 3.2. Assessment of Sexual Behavior and Sperm Quality

At baseline, all rats displayed MSB. Six months after CR, 100% still performed MSB compared to the CR groups after 12 months, when only 33% showed this behavior (two of six). Interestingly, at 24 months old, none of the rats in the control group performed MSB.

With respect to the parameters of MSB, findings show that the mount latency, number of mounts, and intromission latency decreased significantly at 6 months of CR in the 15% and 35% groups compared to the control group (*p* < 0.01, Table 2). The number of ejaculations and the hit rate are parameters that showed an adequate execution of the MSB; these parameters showed a significant increase compared to control group (*p* < 0.01, Table 2). In the control group, we observed a significant increase in the parameters of MSB recorded when compared to the basal values (*p* < 0.01, Table 2).

### 3.3. Sperm Quality

For the parameter of sperm motility, age significantly reduced this parameter in all groups (*p* < 0.01), reaching the lowest values up to 12 months of CR. In the CR 15% and 35% groups after 6 and 12 months of CR, a significant increase was observed when compared to the control group (*p* < 0.01, Table 3). This same fact was observed when compared with the basal value (*p* < 0.05, Table 3).

Regarding vitality and sperm count, after 12 months of CR, a significant decrease was observed in all groups when compared to the basal values (*p* < 0.01. Table 3), and no differences were observed between CR groups 15% and 35% when compared to the control group for vitality but not for sperm count (*p* < 0.05, Table 3). It is important to remark that the fragmentation was not affected due to age or by CR regarding basal values (*p* < 0.05, Table 3).

### 3.4. Metabolic Parameters

CR modified the concentrations of the hormones that participate in the metabolism after 6 and 12 months of CR at 15% and 35% and thus yielded a significant reduction of plasma in glucose, leptin, insulin, glucagon, and estradiol levels compared to the control groups (*p* < 0.01, Table 4). Measurements of plasma T levels showed a significant increase in both CR 15% and 35 groups (*p* < 0.01, Table 4). Note that the 6- and 12-month control groups had significant increases of plasma insulin and estradiol levels and decreased plasma testosterone levels compared with the basal values (*p* < 0.01, Table 4).

Regarding plasma prolactin levels, a significant increase was only observed in the control group after 12 months of experimentation (*p* < 0.01, Table 4).

### 3.5. *Testicular Apoptosis Measured by Annexin V Assay in Seminiferous Tubules*

**Figure 3:**
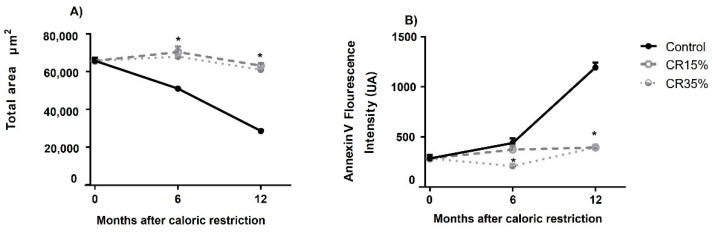
Effect of CR on the total area and apoptosis of testicular tubules in aged male Wistar rats. The values obtained show mean ± SE of the total area of the seminiferous epithelium tubules (**A**) and the fluorescence intensity of the protein annexin V as a pre-apoptotic marker in seminiferous epithelium tubules (**B**) in rats with CR at 15% and 35% for 6 and 12 months of CR. * Indicates significant differences between the CR 15% and 35% vs. the control group, *p* < 0.01.

The area of the seminiferous tubules was affected in the control group, but observations showed that after 6 and 12 months of CR, the CR 15% and CR 35% groups conserved this area with respect to the basal value. Interestingly, the area of the seminiferous tubules decreased 1.2-fold at 6 months and 2.3-fold at 12 months in the control group compared to the CR groups (*p* < 0.01, Figure 3A). In the case of annexin V, no changes were seen in the CR 15% or 35% groups after 6 or 12 months of CR with respect to the basal value, but in the control group, the fluorescence intensity of annexin V increased significantly at 6 and 12 months (*p* < 0.01, Figure 3B).

## 4. Discussion

Our results reveal the benefits of chronic and moderate CR during the aging process in rodents by (i) increasing life expectancy and (ii) maintaining the body weight of the restricted aged rats. Overall, the metabolic profile of the CR rats provides a broad panorama of the energy activity, expenditure, and storage for the correct functioning of individuals [37]. Our findings showed that glucose, insulin, and leptin levels decreased in aged rats subjected to CR for 6 or 12 months. In contrast, these variables were increased in the control rats. These findings confirm and extend previously published, similar experimental paradigms in rodents showing that long-term CR maintains glucose tolerance, improving the secretion of insulin and leptin to prolong well-being during aging. This finding is important because it has been reported that as animals age, glucose levels remain high due to alterations in insulin and glucagon concentrations since the aging process is marked by a deterioration in α and β pancreatic cells [38], increased visceral adiposity, and reduced physical activity, among other phenomena [37,39]. With respect to adiponectin, plasma levels remained constant up to 12 months of CR, suggesting correct glucose metabolism, similar to previously reported [39].

After 12 months of CR, we observed that T concentrations in the rats were similar to those of young adult animals aged 3 months as has been previously published [40]. This effect could be due to the beneficial effects of CR through improved cellular functioning that activates physiological pathways for hormonal secretion from the HPG axis. Our data are supported by previous report [32], where Wistar rats under CR maintained their T levels. This also occurred in experiments with *Macaca mulata* subjected to CR at 30%, where improved functionality of the HPG axis and stable T levels were found compared to a control group [41]. This indicates that aromatase activity is necessary for the aromatization of T to E_2_, which increases its concentration during the aging process [42]. In contrast, E_2_ levels in the control group were increased, likely due to high aromatase activity in animals with greater body weight [42]. In other hand, prolactin has been reported to participate in controlling masculine behavior [20], and also, an increase in its levels can inhibit MSB during aging [23,43]. Our results demonstrate that during aging, the control rats presented a significant increase in prolactin levels as has been previously reported [26].

Based in our understanding in the field, the main contribution of these findings consists in demonstrating that chronic and moderate CR during aging prolongs the reproductive function in aged rats. This fact is support by several studies that have demonstrated the existence of a direct correlation between T concentrations and MSB such that a deficit of T affects MSB even in young adult animals [30,32,44,45]. However, correct performance of MSB requires the synergic activity of T and E_2_ because an increase continued may generate feminization of the brain and increase latencies during MSB tests [46]. Our study found that control rats presented increased E_2_ accompanied by extended latencies on MSB tests. These results demonstrate a partial inactivation of MSB during aging in control rats. Significantly, CR can prevent this process because reduced E_2_ concentrations and improved some parameters of MSB after 6 months of CR in aged rats.

Studies with rats have shown that latencies in mount, intromission, ejaculation, and post-ejaculatory interval are increased after 11 months old, and these latencies are more prolonged at 24 months, when ejaculations decrease [26,28]. Our study found that at 18 months old, the control rats presented an increase in ejaculation latency. These observations allow us to affirm that MSB during aging could be improved after 6 months of CR at 15% and 35%. These observations are supported by a previous report [32], which found that young rats with CR at 25% had enhanced MSB due to reduced intromission and ejaculation latencies.

We observed that sperm motility decreased in the control group after 18 months old, but it was observed in the CR animals. This suggests better cellular functioning that may result from the concentrations of T. Studies with *Macaca mulata* using CR at 30% showed that sperm motility and vitality remained stable as well as T levels [41,47]. These results strengthen our data on sperm motility and vitality, which remained optimal for the CR groups up to 18 months old. However, aging leads to a decrease in sperm vitality even with CR at 24 months old.

Analyses of DNA fragmentation in the sperm are still in an exploratory stage, so the specific mechanism involved has not been clarified [48]. However, it is well known that obesity and aging potentiate this process due to an increase of reactive oxygen species (ROS) [49,50]. In the present study, we did not observe DNA fragmentation in the sperm even under CR treatment.

In another aspect, the increase in apoptosis associated with aging is responsible for the deterioration of testicular functions [51,52]. In this study, we found that the aged rats in the control group showed reduced T levels in the area of the seminiferous tubules with an increase in testicular apoptosis after 18 months old. These data concur with the report earlier [53]; this study found an increase in testicular apoptosis between 12 and 24 months old in rats. The correlation of the expression of annexin V with apoptotic markers in the sperm of men aged over 40 years old has been previously reported [54]. Furthermore, the increase in those markers was found to be directly proportional to the age of the patients [55,56,57]. Another study analyzed sperm apoptosis based on the externalization of serine phosphatidyl, quantified by annexin V, which was increased in men aged over 40 years old [58]. Our CR rats at 15% and 35% did not show changes in testicular apoptosis; however, in the control group, an increase in annexin V was observed.

## 5. Conclusions

The present study demonstrates the beneficial effects of chronic and moderate CR for 6 months on MSB in aged rats. This benefit is dependent on maintaining the animals’ metabolic and hormonal profile. We can, therefore, affirm that CR is an efficacious strategy to prolong certain processes related to reproductive health.

## Figures and Tables

**Table 1:** Effect of CR on body weight in aged male Wistar rats.

		6 Months after CR			12 Months after CR		
	Basal	Control	CR 15%	CR 35%	Control	CR 15%	CR 35%
Body Weight	541.6 ± 6.7	640.2 ± 10.6 ^#^	447.0 ± 19.6 *	437.7 ± 7.2 *	630.3 ± 15.3 ^#^	443.2 ± 15.3 *	446.7 ± 17.0 *

The values obtained show mean ± SE body weight in rats with CR at 15% and 35% for 6 and 12 months of CR. * Indicates significant differences between the 15% and 35% CR groups vs. the control group, *p* < 0.01; ^#^ Indicates significant differences between the control groups after 6 and 12 months of CR vs. the basal value, *p* < 0.05.

**Table 2:** Effect of CR on MSB in aged male Wistar rats.

		**After 6 Months of CR**		
	Basal	Control	CR 15%	CR 35%
Mount latency (s)	56.5 ± 7.37	200.0 ± 47.70 ^#^	83.0 ± 55.33 *	40.83 ± 7.50 *
Number of mounts	3.4 ± 0.77	5.5 ± 1.25	4.16 ± 1.35 *	3.16 ± 0.47 *
Intromission latency (s)	73.6 ± 52.5	142.5 ± 62.08 ^#^	78.0 ± 56.5 *	67.5 ± 28.03 *
Number of intromissions	8.5 ± 0.43	14.5 ± 1.89 ^#^	17.8 ± 2.44 ^#^	14.0 ± 1.61 ^#^
Ejaculation latency (s)	521.0 ± 145.0	624.0 ± 235.0	738.0 ± 165 ^#^	638.0 ± 98.0
Post-ejaculatory interval (s)	443.3 ± 103.4	414.8 ± 155.4	670.8 ± 203.6	442.0 ± 48.13
Number of ejaculations	2.3 ± 0.2	1.0 ± 0.0 ^#^	2.0 ± 1.0	3.0 ± 1.0 *
Hit rate	0.77 ± 0.26	0.67 ± 0.007 ^#^	0.79 ± 0.033 *	0.82 ± 0.25 *

The values obtained show mean ± SE (*n* = 6 per group) for the copulatory parameter of rats with CR at 15% or 35% for 6 months. * Indicates significant differences between the 15% and 35% CR groups vs. the control group, *p* < 0.01; ^#^ Indicates significant differences between the control, 15% and 35% CR groups vs. the basal value, *p* < 0.01.

**Table 3:** Effect of CR on sperm quality in aged male Wistar rats.

		After 6 Months of CR			After 12 Months of CR		
	Basal	Control	CR 15%	CR 35%	Control	CR 15%	CR 35%
Motility (%)	64.0 ± 0.9	43.8 ± 0.9	50.0 ± 2.0 *^#^	56.6 ± 0.8 *^#^	25.3 ± 2.0 ^#^	40.2 ± 0.4 *^#^	43.0 ± 2.5 *^#^
Viability (%)	89.0 ± 2.1	80.3 ± 0.9	81.1 ± 1.6	86.0 ± 1.6	44.1 ± 4.7 ^#^	50.8 ± 6.9 ^#^	55.0 ± 8.4 ^#^
Sperm count (10^6^/mL)	245.0 ± 16.9	220.0 ± 16.6	257.0 ± 8.3	245.0 ± 12.7	148.2 ± 6.2 ^#^	218.0 ± 15.2 *	178.0 ± 9.0 *
Fragmentation (%)	96.4 ± 0.3	97.0 ± 0.4	97.0 ± 0.3	97.0 ± 0.2	96.0 ± 0.3	96.0 ± 0.2	96.0 ± 0.3

The values obtained show mean ± SE (*n* = 6 per group) for the parameters of sperm quality for rats with CR at 15% and 35% for 6 and 12 months. * Indicates significant differences between the 15% and 35% CR groups vs. the control group, *p* < 0.01; ^#^ Indicates significant differences between the 15% and 35% CR groups vs. the basal value, *p* < 0.05.

**Table 4:** Effect of CR on metabolic parameters in aged male Wistar rats.

		After 6 Months of CR			After 12 Months of CR		
	Basal	Control	CR 15%	CR 35%	Control	CR 15%	CR 35%
Glucose (mmol/L)	8.87 ± 0.52	11.88 ± 0.42	6.17 ± 1.23 *	5.72 ± 1.12 *	11.90 ± 0.92	6.07 ± 1.22 *	5.22 ± 0.99 *
Leptin (pg/mL)	2680.0 ± 398.0	3004.0 ± 341.0	773.0 ± 114.0 *	481.0 ± 87.0 *	1427.0 ± 119	466.0 ± 559.0 *	336.0 ± 67.0 *
Adiponectin (ng/mL)	12.18 ± 0.23	12.60 ± 0.80	13.80 ± 0.69	12.30 ± 0.20	10.80 ± 0.18	12.27 ± 0.75	11.60 ± 1.08
Insulin (ng/mL)	0.719 ± 0.11	1.52 ± 0.16	0.532 ± 0.79 *	0.559 ± 0.075 *	4.371 ± 0.56 ^#^	0.513 ± 0.12 *	0.650 ± 0.58 *
Glucagon (pg/mL)	199.0 ± 22.0	237.0 ± 55.0	56.0 ± 8.9 ^#^	63.0 ± 7.1 ^#^	161.0 ± 16.0	74.0 ± 11.0 ^#^	36.0 ± 6.3 ^#^
Testosterone (ng/mL)	1.95 ± 0.04	0.57 ± 0.03 ^#^	1.75 ± 0.14 *	2.10 ± 0.15 *	0.17 ± 0.02 ^#^	1.47 ± 0.07 *	1.17 ± 0.08 *
Estradiol (pg/mL)	1.68 ± 0.03	1.99 ± 0.05 ^#^	1.80 ± 0.03	1.78 ± 0.04 *	1.96 ± 0.06 ^#^	1.76 ± 0.03 *	01.74 ± 0.04 *
Prolactin (pg/mL)	65.0 ± 1.56	80.0 ± 5.07	69.0 ± 4.60	71.0 ± 6.01	92.0 ± 13.16 ^#^	65.0 ± 3.75	66.0 ± 3.63

The values obtained show mean ± SE (*n* = 6 per group) for the metabolic and sexual hormones of animals with CR at 15% and 35% for 6 and 12 months. * Indicates significant differences between the 15% and 35% CR groups vs. the control group, *p* < 0.01; ^#^ Indicates significant differences between the 15% and 35% CR groups vs. the basal value, *p* < 0.01.
